# Malnutrition and Risk of Mortality in Ischemic Stroke Patients Treated With Intravenous Thrombolysis

**DOI:** 10.3389/fnagi.2022.834973

**Published:** 2022-02-21

**Authors:** Haiyan Tang, Fan Gong, Hongquan Guo, Zheng Dai, Jun Wang, Bin Liu, Tingting Li, Xianbiao Tang, Junru Dong, Song Pan, Mingzhe Wang, Yan Sun, Baofeng Qin, Jingsi Zhang, Xuyin Zhu, Jun Tian, Zhimin Fei, Gendi Lu, Dezhi Liu

**Affiliations:** ^1^Department of Neurology, Shuguang Hospital Affiliated to Shanghai University of Traditional Chinese Medicine, Shanghai, China; ^2^Institute of Neurology, Shuguang Hospital Affiliated to Shanghai University of Traditional Chinese Medicine, Shanghai, China; ^3^Department of Neurology, Jinling Hospital, Southern Medical University, Nanjing, China; ^4^Department of Neurology, The Affiliated Wuxi People’s Hospital of Nanjing Medical University, Wuxi, China; ^5^Department of Rehabilitation Medicine, Puer Hospital of Traditional Chinese Medicine, Puer, China; ^6^Changzhou Sports Medical Research Institute, Changzhou, China; ^7^Department of Medical Imaging, Shanxi Medical University, Taiyuan, China; ^8^Department of Nursing, Shuguang Hospital Affiliated to Shanghai University of Traditional Chinese Medicine, Shanghai, China; ^9^Department of Neurosurgery, Shuguang Hospital Affiliated to Shanghai University of Traditional Chinese Medicine, Shanghai, China

**Keywords:** malnutrition, mortality, thrombolytic therapy, stroke, biomarker

## Abstract

**Background and Purpose:**

Malnutrition is highly prevalent in ischemic stroke patients. We aimed to investigate whether malnutrition indexes may be useful in predicting mortality at 90 days in ischemic stroke patients treated with intravenous thrombolysis.

**Methods:**

We retrospectively analyzed consecutive patients who underwent thrombolytic therapy at three comprehensive stroke centers. Malnutrition was assessed using the controlling nutritional status (CONUT) score, geriatric nutritional risk index (GNRI), and prognostic nutritional index (PNI).

**Results:**

Of 979 patients (mean age, 66.8 years; males, 63.6%) included in this study, 91 (9.3%; 95% confidence interval [CI]: 8.4–10.2%) died at 3-month follow up. According to the CONUT, GNRI, and PNI scores, 9.9, 33.7, and 7.0% of patients were moderately or severely malnourished, respectively; 64.0% were at least mildly malnourished by at least 1 malnutrition index. In the multivariate regression model after adjusting for potential confounders, malnutrition (severe risk versus normal nutritional status) was significantly associated with an increased risk of mortality for CONUT scores (adjusted odds ratio [OR] 16.16, 95%CI, 7.86-67.11; *P* < 0.001), GNRI scores (adjusted OR 9.82, 4.10-23.51; *P* < 0.001) and PNI scores (adjusted OR 12.74, 5.56-29.19; *P* < 0.001). Similar results were found when the malnutrition scores were analyzed as continuous variables. Adding the three malnutrition indexes to models containing conventional risk factors significantly improved risk reclassification for 3-month mortality.

**Conclusion:**

Our study showed that malnutrition may be associated with a higher risk of mortality at 3 months in ischemic stroke after intravenous thrombolysis.

## Introduction

Intravenous thrombolysis (IVT) is efficacious and safe in acute ischemic stroke patients (Powers et al., 2019). IVT could improve the odds of a favorable outcome after acute ischemic stroke when delivered within 4.5 h of symptom onset, irrespective of age and stroke severity, and an increased risk of hemorrhagic transformation (Emberson et al., 2014; Whiteley et al., 2016). Nevertheless, a pre-specified meta-analysis of individual patient data from 6,756 patients treated with thrombolytic therapy in nine randomized trials found a 90-day mortality rate of 17.9% (Emberson et al., 2014). Also, the 90-day mortality is not significantly modified by thrombolytic therapy, despite a tendency toward reduced mortality in those who are treated within 1 h (Lees et al., 2010; Emberson et al., 2014). Determining the predictors related to mortality may be helpful to identify patients who might benefit from intensive management and improve long-term outcomes after IVT.

It has been reported that Malnutrition is associated with poor prognosis in a variety of diseases, such as heart failure, acute coronary syndrome, as well as ischemic stroke (Amare et al., 2015; Raposeiras Roubín et al., 2020; Yuan et al., 2021). The prevalence of malnutrition risk in stroke patients ranged widely from 6.0 to 62%, according to a systematic review consisting of 18 studies (Foley et al., 2009). Compared to other risk factors, malnutrition has the advantage that it is a modifiable clinical characteristic on which physicians could act (Freeman et al., 2017). Several objective nutritional tools were performed for evaluating malnutrition risk, including the controlling nutritional status (CONUT) score (Ignacio de Ulíbarri et al., 2005), geriatric nutritional risk index (GNRI) (Bouillanne et al., 2005), and prognostic nutritional index (PNI) (Buzby et al., 1980). Recently, the three malnutrition scores, which can be easily calculated from objective variables, have been validated in predicting the prognosis of cardiovascular diseases and ischemic stroke (Raposeiras Roubín et al., 2020; Yuan et al., 2021; Zhang et al., 2021). Using the three objective malnutrition indexes, data from Third China National Stroke Registry found that malnutrition risk in ischemic stroke patients was linked to a higher risk of long-term death and major disability (Zhang et al., 2021). However, the prognostic value of nutritional status on mortality in patients treated with IVT has not been adequately addressed.

This study aimed to determine the effect of the three malnutrition scores mentioned above as continuous and as categorized variables (normal, mild, moderate, and severe malnutrition risk) on 90-day mortality in a large population of IVT-treated stroke patients.

## Materials and Methods

### Study Population

This study was a retrospective analysis of a prospective registry from three stroke centers in China (Shuguang Hospital between January 2016 and December 2020, Jinling Hospital, and Wuxi People’s Hospital between April 2014 and November 2020). We enrolled patients with acute ischemic stroke who underwent IVT. Patients were excluded from this study if they meet these criteria: (1) age < 18 years old; (2) had incomplete follow-up; (3) had no measurement of variables required to calculate the malnutrition risk during hospitalization; (4) had a history of chronic lymphocytic leukemia, lymphoma, other malignant tumors, as well as active or chronic inflammatory disorders. To keep the homogeneity of the enrolled patients, we also excluded patients treated with endovascular treatment after IVT. This study was approved by the ethics committee of each participating stroke center.

### Baseline Assessments

Demographic characteristics and vascular risk factors, including hypertension, diabetes mellitus, hyperlipidemia, coronary artery disease, and smoking were recorded after admission. Stroke severity was assessed by a certified neurologist using the National Institutes of Health Stroke Scale (NIHSS) (Goldstein and Samsa, 1997) on admission. The stroke subtype was classified according to TOAST (Trial of Org 10172 in Acute Stroke Treatment) criteria (Adams et al., 1993). IVT-related information (onset to treatment time and symptomatic intracranial hemorrhage [sICH]) was collected. sICH was defined according to the criteria of the European Cooperative Acute Stroke Study II (ECASS-II) (Hacke et al., 1998). Laboratory data including lipid profiles, albumin, blood glucose, hypersensitive C-reactive protein (Hs-CRP), and blood cell counts were measured within 24 h after admission.

### Nutritional Screening Scores

All patients were screened for malnutrition risk using the CONUT scores, GNRI scores, and PNI scores. Three variables (including serum albumin, cholesterol, and total lymphocyte count) were comprised of CONUT scores to evaluate nutritional risk in hospitalized patients. A CONUT score of 0–1 was defined as normal nutrition; scores of 2–4, 5–8, and 9–12 indicated mild, moderate, and severe malnutrition. The GNRI was calculated as (1.489 × serum albumin [g/L] + 41.7 × present weight [kg]/ideal body weight [kg]). Ideal body weight was calculated according to the Lorenz formulas: height (cm)-100-([height (cm)−150]/4) for men and height (cm)-100- ([height (cm) −150]/2.5) for women. While the current weight exceeded ideal body weight, the present weight in kilograms/ideal body weight was set as 1. The GNRI scores ≥ 100, 97.50–99.99, 83.50–97.49, and < 83.50 were considered as normal, mild, moderate, and severe malnutrition risk, respectively. The PNI was calculated using the formula: 10 × serum albumin concentrations (g/dL) + 0.005 × lymphocyte count (mm^3^). The PNI scores > 38, 35–38, and < 35 indicated normal, moderate, and severe malnutrition, respectively. The detailed scoring criteria of the malnutrition scores were presented in Supplementary Table 1.

### Outcomes and Follow-Up

Patients were followed up at 3 months after stroke onset by outpatient visit or telephone interview. The endpoint was defined as mortality, which was collected from their relatives, medical records, death certificates, or other available data.

### Statistical Analysis

Continuous variables are presented as mean (standard deviation) or median (interquartile range). Categorical variables were presented as numbers (percentage). Continuous variables were analyzed using the *t*-student test or Mann–Whitney *U* test. Categorical variables were analyzed using the Chi-square test or Fisher’s exact test as appropriate. Correlation between two continuous parameters was evaluated with Spearman correlation coefficient. The multivariate logistic regression model with a forward procedure was used to evaluate the associations between each of all the three malnutrition scores and mortality at 3 months, adjusting for age, gender, coronary heart disease, baseline NIHSS scores, sICH, stroke subtypes, baseline blood glucose, and Hs-CRP levels. Odds ratios (OR) with 95% confidence intervals (CIs) for each of the malnutrition scores were finally calculated. The spline regression model was performed to provide more precise estimates and explore the shape of the association between malnutrition indexes and mortality, fitting a restricted cubic spline function with three knots (at the 5th, 50th, and 95th percentiles). The receiver operating characteristic (ROC) curve analysis was performed to assess the predictive value of the 3 malnutrition scores. In addition, the net reclassification index (NRI) and integrated discrimination improvement (IDI) were calculated to assess the predictive value of adding malnutrition scores to the conventional risk factors model. A two-sided *P* < 0.05 was considered statistically significant. Statistical analysis was performed using R statistical software version 3.6.1 (R Foundation, Vienna, Austria) and SPSS 22.0 (SPSS Inc., Chicago, IL, United States).

## Results

### Baseline Characteristics

A total of 1,040 acute ischemic stroke patients treated with IVT were screened. Among them, 37 patients without data of 90-day mRS, 21 patients had no measurement of variables required to calculate the malnutrition risk, and 3 patients had a history of leukemia or lymphoma. Finally, 979 acute ischemic stroke patients were included for the analysis. The average age of these patients was 66.8 years. Of these patients, 63.6% were men. Among these patients, 69.1% had hypertension, 24.0% had diabetes mellitus, 12.4% had hyperlipidemia, and 9.3% had a history of coronary heart disease. The median NIHSS was 6 (IQR 3–12) at baseline. The mean onset to needle time was 132.0 min. More data on the baseline characteristics of the study sample were shown in Table 1.

**TABLE 1:** Demographics and baseline characteristics stratified by clinical outcome.

Variables	All patients, *n* = 979	Death, *n* = 91	Survival, *n* = 888	*P* value
**Demographic characteristics**				
Age, years	66.8 ± 13.2	72.3 ± 11.8	66.3 ± 13.2	< 0.001
Male, *n* (%)	623 (63.6)	59 (64.8)	564 (63.5)	0.803
**Medical history**				
Hypertension, *n* (%)	676 (69.1)	68 (74.7)	608 (68.5)	0.219
Diabetes mellitus, *n* (%)	235 (24.0)	23 (25.3)	212 (23.9)	0.766
Hyperlipidemia, *n* (%)	121 (12.4)	14 (15.4)	107 (12.0)	0.357
Coronary heart disease, *n* (%)	91 (9.3)	14 (15.4)	77 (8.7)	0.036
Currently smoking, *n* (%)	439 (44.8)	39 (42.9)	400 (45.0)	0.689
Currently drinking, *n* (%)	307 (31.4)	31 (34.1)	276 (31.1)	0.559
**Clinical data**				
Systolic blood pressure, mmHg	132.9 ± 23.2	135.9 ± 24.7	132.6 ± 23.0	0.195
Diastolic blood pressure, mmHg	84.6 ± 14.1	82.9 ± 13.2	84.8 ± 14.1	0.212
Onset to treatment time, min	132.0 (105.0, 170.0)	137.0 (113.0, 167.0)	131.0 (105.0, 170.0)	0.323
Baseline NIHSS, score	6.0 (3.0, 12.0)	14.0 (8.0, 21.0)	6.0 (3.0, 11.0)	< 0.001
sICH, *n* (%)	67 (6.8)	20 (22.0)	47 (5.3)	< 0.001
**Stroke etiology, *n* (%)**				** < 0.001**
Large artery atherosclerosis	320 (32.7)	35 (38.5)	285 (32.1)	
Cardio-embolism	183 (18.7)	38 (41.8)	145 (16.3)	
Small vessel occlusion	281 (28.7)	6 (6.6)	275 (31.0)	
Other determined etiology	81 (8.3)	4 (4.4)	77 (8.7)	
Undetermined etiology	114 (11.6)	8 (8.8)	106 (11.9)	
Malnutrition indexes				
CONUT, score	1.0 (0, 3.0)	3.0 (1.0, 6.0)	1.0 (0, 2.0)	< 0.001
**CONUT categorical, n (%)**				** < 0.001**
Normal	533 (54.4)	28 (30.8)	505 (56.9)	
Mild	349 (35.6)	30 (33.0)	319 (35.9)	
Moderate	81 (8.3)	25 (27.5)	56 (6.3)	
Severe	16 (1.6)	8 (8.8)	8 (0.9)	
GNRI, score	100.8 (95.6, 103.9)	94.5 (85.6, 102.6)	101.1 (96.3, 104.1)	< 0.001
**GNRI categorical, *n* (%)**				** < 0.001**
Normal	537 (54.9)	31 (34.1)	506 (57.0)	
Mild	112 (11.4)	6 (6.6)	106 (11.9)	
Moderate	286 (29.2)	40 (44.0)	246 (27.7)	
Severe	44 (4.5)	14 (15.4)	30 (3.4)	
PNI, score	48.9 (44.7, 53.4)	44.9 (37.0, 49.5)	49.2 (45.2, 53.9)	< 0.001
**PNI categorical, *n* (%)**				** < 0.001**
Normal	911 (93.0)	65 (71.4)	846 (95.3)	
Moderate	26 (2.7)	9 (9.9)	17 (1.9)	
Severe	42 (4.3)	17 (18.7)	25 (2.8)	
Laboratory data				
Baseline blood glucose, mmol/L	7.9 ± 3.5	8.7 ± 4.5	7.8 ± 3.5	0.021
Hs-CRP, mg/L	1.3 (0.5, 2.5)	1.7 (0.7, 3.9)	1.3 (0.5, 2.4)	0.004
Albumin, g/L	39.4 ± 5.3	36.0 ± 6.7	39.8 ± 5.0	< 0.001
Lymphocyte count, 10^9^/L	1.8 (1.3, 2.4)	1.5 (0.9, 2.0)	1.8 (1.3, 2.4)	< 0.001
Total cholesterol, mmol/L	4.1 ± 1.3	4.4 ± 1.2	4.6 ± 1.2	0.084
Triglyceride, mmol/L	1.3 (0.9, 1.9)	1.2 (0.9, 1.8)	1.3 (0.9, 1.9)	0.256
Low density lipoprotein, mmol/L	2.6 (2.1, 3.3)	2.6 (1.9, 3.2)	2.6 (2.1, 3.3)	0.156
High density lipoprotein, mmol/L	1.3 ± 0.6	1.3 ± 0.5	1.3 ± 0.6	0.966

*CONUT, controlling nutritional status score; GNRI, geriatric nutritional risk index; Hs-CRP, hypersensitive C-reactive protein; NIHSS, national institute of health stroke scale; PNI, prognostic nutritional index; sICH, symptomatic intracranial hemorrhage. The bold values means difference of mortality rates between the different stroke etiology and malnutrition degree using the Chi-square test.*

### Prevalence of Malnutrition

All malnutrition scores were related to each other (CONUT vs. GNRI, *r* = −0.643; CONUT versus. PNI, *r* = −0.751; GNRI versus. PNI, *r* = 0.771, all *P* < 0.001). Only 6.9% of patients were defined as malnourished (any degree of malnutrition) by all three malnutrition indexes, and 36.0% were not malnourished by any indexes. The prevalence of patients with malnutrition varied from 7.0% with the PNI, to 45.1% with the GNRI, and 45.6% with the CONUT. According to CONUT, GNRI, and PNI scores, there are 9.9, 33.7, and 7.0% of patients had moderate to severe malnutrition risk, respectively. The baseline data stratified by the nutritional status was demonstrated in Supplementary Table 2.

### Association Between Malnutrition Scores and Mortality

During the 3-month follow-up, ninety-one (9.3%) patients died. Compared with survivors, patients died at 3 months were older (72.3 ± 11.8 vs. 66.3 ± 13.2 years, *P* < 0.001), more likely to have coronary heart disease (15.4% vs. 8.7%; *P* = 0.036), sICH (22.0% vs. 5.3%, *P* < 0.001) and cardioembolic stroke (41.8% vs. 16.3%; *P* < 0.001), and had a higher baseline NIHSS scores (median 14.0 vs. 6.0; *P* < 0.001), baseline blood glucose (mean 8.7 ± 4.5 vs. 7.8 ± 3.5 mmol/L; *P* = 0.021) and Hs-CRP levels (median 1.7 vs. 1.3 mg/L; *P* = 0.004). In addition, on univariate analysis, the 3 malnutrition indexes were associated with mortality (all *P* < 0.001).

**FIGURE 1:**
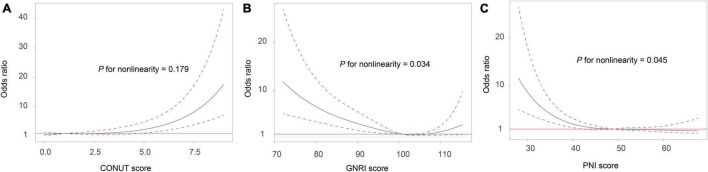
Association between malnutrition and risk of mortality in ischemic stroke patients receiving thrombolytic therapy. Malnutrition was defined by the CONUT score **(A)**, GNRI score **(B)** and PNI score **(C)**. Odds ratios and 95% confidence intervals derived from restricted cubic spline regression, with knots placed at the 5th, 50th, and 95th percentiles of the distribution of the 3 malnutrition indexes. The reference point for malnutrition score is the midpoint of the reference group from the categorical analysis. The odds ratio was adjusted for age, gender, coronary heart disease, baseline NIHSS score, symptomatic intracranial hemorrhage, stroke subtypes, baseline blood glucose, and hypersensitive C-reactive protein levels. CONUT, controlling nutritional status score; GNRI, geriatric nutritional risk index; PNI, prognostic nutritional index.

In multivariate regression analysis after adjusted for potential confounders, the 3 malnutrition indexes were significantly related to 3-month mortality (adjusted OR, 1.38, 95%CI, 1.25–1.53; *P* < 0.001), GNRI score (adjusted OR, 0.94, 95%CI, 0.91–0.96; *P* < 0.001), and PNI score (adjusted OR, 0.92, 95%CI, 0.89–0.95; *P* < 0.001). The observed association remained significant when the malnutrition scores were analyzed as a categorical variable (Table 2). The multiple-adjusted spline regression model further confirmed that worsening malnutrition status was associated with an increased risk of mortality regardless of the malnutrition index used (Figure 1).

**TABLE 2:** Logistic regression analyses of the three malnutrition indexes to predict mortality at 3 months after intravenous thrombolysis.

Variables	Unadjusted model, OR (95% CI)	*P* value	Model 1, OR (95% CI)	*P* value	Model 2, OR (95% CI)	*P* value
CONUT, per 1-point increment	1.40 (1.29–1.53)	< 0.001	1.37 (1.26–1.50)	< 0.001	1.38 (1.25–1.53)	< 0.001
CONUT categorical		< 0.001		<0.001		< 0.001
Normal	Reference		Reference		Reference	
Mild	1.69 (0.99–2.89)		1.55 (0.91–2.66)		1.27 (0.71–2.27)	
Moderate	8.05 (4.39–14.75)		6.47 (3.48–12.03)		6.03 (2.94–12.37)	
Severe	18.04 (6.30–51.61)		18.02 (6.11–53.12)		16.16 (7.86–67.11)	
GNRI, per 1-point increment	0.94 (0.92–0.96)	< 0.001	0.95 (0.92–0.96)	< 0.001	0.94 (0.91–0.96)	< 0.001
GNRI categorical		< 0.001		<0.001		< 0.001
Normal	Reference		Reference		Reference	
Mild	0.92 (0.38–2.27)		0.86 (0.35–2.13)		0.74 (0.27–2.03)	
Moderate	2.65 (1.62–4.35)		2.16 (1.29–3.60)		1.98 (1.11–3.52)	
Severe	7.62 (3.67–15.82)		6.13 (2.89–13.04)		9.82 (4.10–23.51)	
PNI, per 1-point increment	0.91 (0.89–0.94)	< 0.001	0.92 (0.89–0.94)	< 0.001	0.92 (0.89–0.95)	< 0.001
PNI categorical		< 0.001		<0.001		< 0.001
Normal	Reference		Reference		Reference	
Moderate	6.89 (2.96–16.06)		5.13 (2.16–12.21)		7.27 (2.64–20.11)	
Severe	8.85 (4.55–17.22)		8.58 (4.34–16.98)		12.74 (5.56–29.19)	

*CI, confidence interval; CONUT, controlling nutritional status score; GNRI, geriatric nutritional risk index; OR, odds ratio; PNI, prognostic nutritional index.*

*Model 1: adjusted for age and gender;*

*Model 2: adjusted for age, gender, coronary heart disease, baseline NIHSS score, symptomatic intracranial hemorrhage, stroke subtypes, baseline blood glucose, and hypersensitive C-reactive protein levels.*

The ROC analysis showed that the CONUT score (AUC, 0.701; 95CI% 0.639-0.763; *P* < 0.001), PNI score (AUC, 0.696; 95CI% 0.634-0.758; *P* < 0.001), and GNRI score (AUC, 0.669; 95CI% 0.601-0.737; *P* < 0.001) are considered sensitive in predicting the mortality in ischemic stroke treated with IVT. Adding three malnutrition scores to a model containing conventional risk factors significantly improved the category-free NRI and IDI for the prediction of mortality (all *P* < 0.05; Table 3).

**TABLE 3:** Reclassification Statistics (95% CI) for mortality by malnutrition indexes among patients with ischemic stroke after intravenous thrombolysis.

Models	category-free NRI		IDI	
	Estimate (95% CI)	*P* value	Estimate (95% CI)	*P* value
**Model 1**				
+ CONUT	0.304 (0.204–0.404)	< 0.001	0.075 (0.046–0.104)	< 0.001
+ GNRI	0.121 (0.043–0.199)	0.002	0.032 (0.016–0.047)	< 0.001
+ PNI	0.262 (0.165–0.359)	< 0.001	0.052 (0.032–0.072)	< 0.001
**Model 2**				
+ CONUT	0.168 (0.052–0.285)	0.005	0.075 (0.040–0.109)	< 0.001
+ GNRI	0.079 (−0.024–0.182)	0.132	0.038 (0.016–0.061)	< 0.001
+ PNI	0.167 (0.078–0.256)	< 0.001	0.044 (0.021–0.067)	< 0.001

*CI, confidence interval; cNRI, category-free net reclassification improvement; CONUT, controlling nutritional status score; GNRI, geriatric nutritional risk index; IDI, integrated discrimination improvement; PNI, prognostic nutritional index.*

*Model 1: adjusted for age and gender;*

*Model 2: adjusted for age, gender, coronary heart disease, baseline NIHSS score, symptomatic intracranial hemorrhage, stroke subtypes, baseline blood glucose, and hypersensitive C-reactive protein levels.*

## Discussion

In this multicenter retrospective study, we observed that the malnutrition prevalence varied from 7.0% using the PNI, to 45.1% using the GNRI, and 45.6% using the CONUT. Malnutrition was significantly associated with a higher risk of mortality, even after adjustment for several potential confounders. Furthermore, adding the three malnutrition indexes to a model containing conventional risk factors significantly improved the risk prediction of mortality.

It is a common condition for ischemic stroke patients to suffer from malnutrition. In our study of ischemic stroke patients treated with IVT, six hundred twenty-seven (64.0%) were diagnosed as malnourished based on the three scores. Moreover, the incidence of moderate to severe malnutrition risk ranged from 7.0 to 33.7%. The prevalence of moderate to severe malnutrition in this cohort was higher than that previously demonstrated in Third China National Stroke Registry (Zhang et al., 2021) and a hospital-based retrospective study in Japan (Naito et al., 2020), as well as similar to that reported in Nanjing Stroke Registry Program (Yuan et al., 2021). This discrepancy of variation in malnutrition prevalence may be attributed to the difference in nutritional screening tools, timing, and populations. In recent years, several studies have investigated the prognostic value of objective scores in stroke patients (Maruyama et al., 2018; Cai et al., 2020; Kang et al., 2020; Naito et al., 2020; Xiang et al., 2020). A double-center retrospective registry study in Japan that included 1,518 subjects with ischemic stroke and measured malnutrition status with the CONUT scores demonstrated that malnutrition was independently related to poor outcomes in the patients with cardioembolic stroke or stroke of other etiologies (Naito et al., 2020). In a prospective registry study that evaluated the nutritional status by GNRI scores, the authors found that severe malnutrition was associated with 90-day poor outcomes (Kang et al., 2020). Maruyama et al. reported that malnutrition, assessed using the GNRI scores, may have a negative impact on long-term clinical outcomes in stroke patients after rehabilitation (Maruyama et al., 2018). In addition, the PNI was found to have a prognostic value in ischemic stroke patients undergoing IVT (Xiang et al., 2020). However, the association of malnutrition with 90-day mortality in ischemic stroke patients after IVT has yet to be determined. Considering that the three objective nutrition scores may be captured different aspects of malnutrition, it might be better to comprehensively evaluate the malnutrition risk of ischemic stroke patients combining the three malnutrition screening tools. Our study confirmed that ischemic stroke patients undergoing IVT with moderate to severe malnutrition risk are at significantly higher risk of 3-month mortality, regardless of which malnutrition indexes were used. Our findings further confirm the importance of assessing the nutritional status of ischemic stroke patients at admission. In addition, in the FOOD trial, a supplemented diet could decrease the risk of death and poor outcomes in stroke patients (FOOD Trial Collaboration, 2003). All these data strongly support the need for clinicians to integrate the identification of malnutrition in their daily practice, which may improve the risk stratification in ischemic stroke patients treated with IVT.

Although the mechanisms by which malnutrition affects mortality in ischemic stroke patients after IVT are unclear, the factors constituting the malnutrition scores might explain this mechanism. Albumin is a multifunctional protein in the ischemic brain that could play a neuroprotective role, such as inhibiting oxidative stress (Ishizaka et al., 2007), suppressing the various cytokines adhesion within post-capillary microcirculation (Belayev et al., 2002), modifying the platelet aggregation (George and Alastair, 1992; Maalej et al., 1999), and transporting the free fatty acids post-ischemia (Rodriguez de Turco et al., 2002). Gao et al. (2021) have reported that lower serum albumin levels are associated with poorer outcomes in patients with reperfusion therapy. In a rat model of arteriolar thrombosis, albumin therapy was found to enhance the effect of thrombolysis on local vascular dynamics (Park et al., 2008). However, a randomized controlled trial did not confirm a clinical benefit of 25% albumin in patients with ischemic stroke (Ginsberg et al., 2013). In patients treated with mechanical thrombectomy, decreased lymphocyte counts are associated with hemorrhagic complications and long-term outcomes (Semerano et al., 2019). Our study did not report a significant association of total cholesterol level with mortality in the univariate analysis. This was also observed in other studies (Yuan et al., 2021), highlighting the importance of variability in total cholesterol level (Kim et al., 2017). Further studies are warranted to comprehensively evaluate the association of total cholesterol level with clinical outcomes in patients undergoing IVT.

Several limitations of the study should be noted. Firstly, the observational nature of this study does not allow to infer causality. Secondly, we did not compare the prognostic value of the 3 malnutrition scores with more comprehensive nutritional assessments, such as the Malnutrition Universal Screening Tool and Nutritional Risk Screening Tool 2002. Thirdly, although we controlled for a considerable number of potential confounders in the multivariate regression analysis, there was also a possibility of residual confounding. Fourthly, this study lacked detailed nutritional information, including dietary intake and weight change after hospital discharge, which may affect the rehabilitation during follow-up. Finally, this is a retrospective study among Chinese ischemic stroke patients; therefore, our results may not be generable to other ethnic populations and geographical regions. Further studies are needed to confirm our findings in different populations.

In summary, our study suggested that malnutrition assessed by CONUT, GNRI, and PNI could be an effective predictor of 90-day mortality of ischemic stroke patients undergoing IVT. Adequate monitoring of nutritional status could help neurologists to identify patients after IVT at increased risk for mortality. Further studies are also warranted to determine the efficacy of nutrition management in IVT patients.

## Data Availability Statement

The raw data supporting the conclusions of this article will be made available by the authors, without undue reservation.

## Ethics Statement

This study was approved by the ethics committees of Shuguang Hospital, Jinling Hospital, and Wuxi People’s Hospital. The ethics committees waived the requirement of written informed consent for participation.

## Author Contributions

DL and GL designed the study and revised the manuscript. HT, FG, HG, ZD, JW, and BL recorded the clinical data. HT and DL carried out data analysis and wrote the manuscript. TL, XT, JD, SP, MW, YS, BQ, JZ, XZ, JT, and ZF suggested the important data analysis. All authors contributed to the article and approved the submitted version.

## Conflict of Interest

The authors declare that the research was conducted in the absence of any commercial or financial relationships that could be construed as a potential conflict of interest.

## Publisher’s Note

All claims expressed in this article are solely those of the authors and do not necessarily represent those of their affiliated organizations, or those of the publisher, the editors and the reviewers. Any product that may be evaluated in this article, or claim that may be made by its manufacturer, is not guaranteed or endorsed by the publisher.
