# Supplementary material for: Malnutrition and Risk of Mortality in Ischemic Stroke Patients Treated With Intravenous Thrombolysis
Source: Front Aging Neurosci. 2022 Feb 21;14:834973. doi: 10.3389/fnagi.2022.834973 (PMC8901046; doi:10.3389/fnagi.2022.834973)
Supplement: Supplementary file 1 [file Table_1.DOCX]

**Supplementary materials**

**Table 1.** Procedures for the evaluation of each nutritional index.

| Malnutrition scoring systems | Risk of Malnutrition | | | |
| --- | --- | --- | --- | --- |
|  | Absent | Mild | Moderate | Severe |
| CONUT, points | 0-1 | 2-4 | 5-8 | 9-12 |
| Albumin, g/dl | ≥ 3.5 | 3.0-3.4 | 2.5-2.9 | < 2.5 |
| Score | 0 | 2 | 4 | 6 |
| Total cholesterol, mg/dl | ≥ 180 | 140-179 | 100-139 | < 100 |
| Score | 0 | 1 | 2 | 3 |
| Lymphocyte count, 10^9^/L | ≥ 1.60 | 1.20-1.59 | 0.80-1.19 | < 0.80 |
| Score | 0 | 1 | 2 | 3 |
| GNRI, points | ≥ 100 | 97.50-9.99 | 83.50-97.49 | < 83.50 |
| Formula | 1.489 x serum albumin (g/L) + 41.7 x (weight in kilograms/ideal weight*) | | | |
| PNI, points | > 38 | - | 35-38 | < 35 |
| Formula | 10 x serum albumin (g/dL) + 0.005 x total lymphocyte count (per mm^3^) | | | |

Abbreviations: CONUT, controlling nutritional status score; GNRI, geriatric nutritional risk index; PNI, prognostic nutritional index.

*Ideal weight was calculated using the Lorenz formulas: height (cm) - 100 - ([height (cm) -150]/4) for men and height (cm) -100 - ([height (cm) -150]/2.5) for women. When current weight exceeded ideal body weight, we set current weight in kilograms/ideal weight = 1.**Table 2.** Demographics and baseline characteristics according to the patients with and without malnutrition.

| Variables | Nonmalnourished by all 3 scores  n = 352 | Any degree of malnutrition by all 3 scores  n = 627 | *P* value |
| --- | --- | --- | --- |
| Demographic characteristics |  |  |  |
| Age, years | 63.4 ± 12.4 | 68.8 ± 13.2 | <0.001 |
| Male, n (%) | 219 (62.2) | 404 (64.4) | 0.489 |
| Medical history |  |  |  |
| Hypertension, n (%) | 242 (68.8) | 434 (69.2) | 0.879 |
| Diabetes mellitus, n (%) | 79 (22.4) | 156 (24.9) | 0.392 |
| Hyperlipidemia, n (%) | 39 (11.1) | 82 (13.1) | 0.362 |
| Coronary heart disease, n (%) | 21 (6.0) | 70 (11.2) | 0.007 |
| Currently smoking, n (%) | 156 (44.3) | 283 (45.1) | 0.805 |
| Clinical data |  |  |  |
| Systolic blood pressure, mmHg | 135.5 ± 23.6 | 131.6 ± 22.9 | 0.019 |
| Diastolic blood pressure, mmHg | 86.1 ± 13.7 | 83.8 ± 14.2 | 0.026 |
| Onset to treatment, min | 132.5 (106.5, 170.0) | 132.0 (105.0, 170.0) | 0.887 |
| Baseline NIHSS, score | 5.0 (3.0, 10.0) | 7.0 (4.0, 13.0) | <0.001 |
| sICH, n (%) | 20 (5.7) | 47 (7.5) | 0.281 |
| Mortality at 3 months, n (%) | 18 (5.1) | 73 (11.6) | 0.002 |
| Stroke etiology, n (%) |  |  | 0.115 |
| Large artery atherosclerosis | 118 (33.5) | 202 (32.2) |  |
| Cardio-embolism | 47 (13.4) | 183 (18.7) |  |
| Small vessel occlusion | 113 (32.1) | 281 (28.7) |  |
| Other determined etiology | 27 (7.7) | 81 (8.3) |  |
| Undetermined etiology | 47 (13.4) | 114 (11.6) |  |
| Laboratory data |  |  |  |
| Total cholesterol, mmol/L | 5.1 ± 1.0 | 4.3 ± 1.3 | <0.001 |
| Triglyceride, mmol/L | 1.5 (1.0, 2.2) | 1.2 (0.9, 1.7) | 0.004 |
| Low density lipoprotein, mmol/L | 2.9 (2.3, 3.5) | 2.6 (1.9, 3.2) | 0.001 |
| High density lipoprotein, mmol/L | 1.3 ± 0.6 | 1.3 ± 0.6 | 0.823 |
| Baseline blood glucose, mmol/L | 7.9 ± 3.6 | 7.8 ± 3.4 | 0.696 |
| Hs-CRP, mg/L | 1.3 (0.5, 2.2) | 1.3 (0.5, 2.7) | 0.012 |
| Albumin, g/L | 42.8 ± 2.9 | 37.4 ± 5.4 | <0.001 |
| Lymphocyte count, 10^9^/L | 2.1 (1.8, 2.7) | 1.5 (1.1, 3.1) | <0.001 |

Abbreviations: Hs-CRP, hypersensitive C-reactive protein; NIHSS, national institute of health stroke scale; sICH, symptomatic intracranial hemorrhage.
